# Evaluating the mental health and well-being of Canadian healthcare workers during the COVID-19 outbreak

**DOI:** 10.1177/08404704211021109

**Published:** 2021-06-08

**Authors:** Jonathan M. P. Wilbiks, Lisa A. Best, Moira A. Law, Sean P. Roach

**Affiliations:** 1 Department of Psychology, 96944 University of New Brunswick, Saint John, New Brunswick, Canada.

## Abstract

During the COVID-19 pandemic, healthcare systems have been under extreme levels of stress due to increases in patient distress and patient deaths. While additional research and public health funding initiatives can alleviate these systemic issues, it is also important to consider the ongoing mental health and well-being of professionals working in healthcare. By surveying healthcare workers working in Canada during the COVID-19 pandemic, we found that there was an elevated level of depressive symptomatology in that population. We also found that when employees were provided with accurate and timely information about the pandemic, and additional protective measures in the workplace, they were less likely to report negative effects on well-being. We recommend that healthcare employers take these steps, as well as providing targeted mental health interventions, in order to maintain the mental health of their employees, which in turn will provide better healthcare at the population level.

## Introduction

During COVID-19, healthcare systems are stressed by increasing numbers of patients in distress and patient deaths as well as new requirements for protection of patients and healthcare workers. Although the issue is systemic, healthcare professionals working during the first wave of COVID-19 faced pandemic-related stressors including depression, anxiety, and Post-Traumatic Stress Disorder (PTSD).^1–5^ Given that symptoms of depression are long-lasting,^6^ pandemic-related psychological distress is likely to persist beyond the active pandemic stages. Our research examined the physical and mental well-being of healthcare workers in Canada during the COVID-19 pandemic and the protective effects that employers can have by instituting safety measures for employees.

## Workplace stressors during pandemics

During the H1N1 and SARS-1 pandemics, healthcare workers reported increased mental health distress.^7,8^ Sirois and Owens^9^ reviewed data from previous pandemics and found that psychological distress was associated with being female, being a nurse, and having contact with infected patients; increased social support, proper training, and appropriate Personal Protective Equipment (PPE) were protective factors. Healthcare workers in a large public hospital in Israel during COVID-19 reported elevated levels of depression and PTSD.^9^ Given the detrimental effects of acute and chronic psychological distress, some countries, including the United Kingdom, have implemented counselling programs to help healthcare staff cope with ongoing pandemic-related stress.^10^

One consequence of long-term psychological distress is the risk of burnout,^11^ which is associated with disconnection from others, depersonalization, and loss of professional motivation. Burnout syndrome, classified by the *International Classification of Diseases 11^th^ Revision* as an occupational phenomenon, occurs when employees are under chronic workplace stress that is properly cared for.^12^ Burnout involves feelings of exhaustion, mental distance, and/or cynicism about one’s job and reduced professional efficacy. Recovery is challenging and often requires an extended leave of absence or a change of workplace, which is undesirable for employees and employers. Although an occupational disorder, having burnout for an extended period often leads to more pervasive issues, including declines in general well-being, increased absenteeism, and clinical depression. In a study conducted during spring 2020, Gilin and colleagues^13^ reported elevated levels of emotional exhaustion and increased cynicism in healthcare workers, which highlights the need for targeted interventions to minimize burnout risk by addressing increased psychological distress during times of stress.

The combination of professional and personal stressors faced by healthcare workers during the first wave of COVID-19 creates a knock-on effect, whereby personal stressors affect performance at work, which stresses the healthcare system, and ultimately affect population-level disease outcomes.^14^ Despite the realization that the mental health of healthcare workers was negatively impacted by COVID-19 and research highlighting the importance of providing ongoing, accessible mental healthcare for healthcare workers,^10,14,15^ few workers sought out mental health support.^2^ The effects of already heavy workloads, adjustments to work conditions, and caregiving responsibilities during the institution of public health measures at a societal level can exacerbate stress in healthcare workers and highlight the importance of preventative measures that target workplace stress.^10^

## Factors that promote wellness during pandemic restrictions

Workplace conditions, including clear communication of public health directives and associated training, have a protective impact on the well-being of healthcare workers during public health crises.^16,17^ Positive feedback from administration,^18^ adequate crisis response training,^19^ and adequate time off^20^ buffer the psychological impact of acute stressors in the healthcare workplace. Further, there is an association between the accurate and timely communication of information and psychological distress in the general population^21^ and in healthcare workers.^22,23^ Provision of adequate PPE^16,17,24,25^ and rapid establishment of infection control procedures^26,27^ are associated with a reduction of negative impacts on medical personnel. Healthcare workers reported that increased infection control protocols were evidence that administrators recognized the severity of the outbreak and were concerned about staff safety, which can result in lower distress.^28,29^ Individuals with greater interpersonal and social support in their workplace were buffered against stress, anxiety, and sleep disruptions during pandemic conditions.^22,30^ These findings have significant implications for the management of the frontline healthcare teams in terms of scheduling, institutional support, and provision of psychiatric services to alleviate distress.^28^

In the LEADS framework,^31^ the Engage Others domain implores leaders to foster the development of others, communicate effectively, build teams, and contribute to the creation of a healthy organization. In the context of this research, it is important to consider effective communication and the creation (or maintenance) of a healthy organization. The provision of clear, accurate, and timely information from leadership is associated with decreased burnout and increased mental well-being. Although the importance of this type of communication is not exclusive to a crisis such as COVID-19, it is essential when employees are under increased workload and workplace stress.

Given the increased psychological distress faced by healthcare workers, our purpose was to identify factors associated with increased distress and inform the establishment of best practices that could be implemented in subsequent waves of the COVID-19 pandemic and during future public health crises. We assessed the general and mental health of healthcare workers by administering questionnaires that measure depression, anxiety, and burnout. Participants also answered questions to assess the response of their workplace and measures implemented to reduce the risk of COVID-19 transmission as well as their satisfaction with these measures and communication from management. We hypothesized that healthcare workers would report increased depression, anxiety, and burnout during the COVID-19 pandemic. We expected to find a negative association between psychological distress and management response, suggesting that distress is lower when healthcare workers perceive that their employer is doing its utmost to protect them.

## Method

### Participants

The survey was completed by 86 Canadian healthcare workers (79 female, 7 male; M_age_ = 38.7 years, SD = 12.0). Occupations and workplaces for participants are displayed in Table 1.

**Table 1.** Occupation and workplace for participants

	Freq.
Occupation	
Technician	27
Registered nurse	19
Personal support worker	5
Pharmacist (or assistant)	4
Family doctor/GP	2
Specialist	1
Nurse practitioner	1
Orderlies	3
Administrative staff	3
Other	21
Workplace	
Hospital/clinic	52
Long-term care facility	10
Private office	2
Hospital and long-term care	10
Other	18

### Materials

Participants completed questionnaires to assess work satisfaction and well-being during the COVID-19 pandemic, including questions to assess protective measures taken in their workplaces. Participants used a 7-point Likert scale to rate their satisfaction with the implementation of COVID-19 safety measures and employer communications as well as measures to assess psychological distress and general well-being. The Patient Health Questionnaire-9 (PHQ-9)^32^ is a 9-item Likert scale measure of depressive symptomatology that includes an item to assess suicidal ideation. The original scale construction was valid and reliable (Cronbach α = .89),^32^ with high reliability in the current study (α = .86). The General Well-Being Questionnaire (GWB)^33^ includes 28 Likert-scale items to measure mental and physical well-being and includes subscales that assess symptoms of burnout (worn out) and anxiety (uptight).^33^ The GWB has high levels of validity and reliability originally (α = .88)^33^ and in our sample (α = .91).

### Procedure

The current study was approved by the research ethics board at the University of New Brunswick. Data collection occurred on-line through Qualtrics between May 30 and June 13, 2020, during which time Canada was nearing the end of the “first wave” of COVID-19, with an average of 933 new cases of COVID-19 on May 30 and an average of 510 new cases on June 13.^34^ Participant recruitment was conducted via advertisements on social media platforms and email mailing lists, specifically targeting healthcare workers. After giving informed consent, participants completed a demographics questionnaire, which was followed by the remainder of the surveys, presented in a randomized order so as to prevent bias caused by completion order.

## Results and discussion

Table 2 presents means and SDs for each measure. The median number of protective measures instituted by workplaces was 7 (M = 6.56, SD = 2.05; see Table 2). The most common measures were increased PPE (93%), health screening at entry (88%), visitor limitations (86%), and required increases in handwashing (86%); 31% of employees could work from home, representing the least common health measure used by management. Most respondents were relatively satisfied with the protective measures that were instituted, with the average satisfaction rating 5.3 (SD = 1.7) on a 7-point Likert scale. Respondents were generally satisfied with the amount of information they received from their employers, with an average satisfaction rating of 5.0 (SD = 1.8). We observed significant positive correlations between number of safety measures and employee satisfaction, *r* = .44, *P* < .001, and communication satisfaction, *r =* .52, *P <* .001, replicating previous research suggesting that employees appreciated safety measures^28,29^ increased employer communication.^16,17^

**Table 2.** Descriptive statistics for each measure

Measure	M	SD
Sum of health measures	6.56	2.05
Satisfaction with protection	5.31	1.74
Satisfaction with information	5.02	1.78
Physical health effects	3.44	1.24
Mental health effects	4.22	0.99
GWB—worn out	12.22	6.45
GWB—uptight	8.40	5.73
PHQ-9	8.00	5.30

Abbreviations: GWB, General Well-Being Questionnaire; PHQ-9, Patient Health Questionnaire-9.

Participants rated how much their physical and mental health were impacted by lifestyle changes caused by the pandemic. Although participants indicated that the impact on their physical health was mild/moderate (M = 3.4, SD = 1.2), they reported greater mental health effects (M = 4.2, SD = 1.0; *t*(85) = 6.70, *P* < .001). The General Well-being Scale revealed burnout scores (M = 15.0, SD = 9.1) similar to norms established by Cox et al.,^33^ which was 14.9 (SD = 9.4), and anxiety scores (M = 10.1, SD = 7.7) that were below the norm of 17.6 (SD = 10.6; Cox et al.^33^). Thus, during this phase of the pandemic, healthcare professionals in this sample did not have increased symptoms of burnout or acute stress, which may be explained by the declining case counts at the time of survey administration. This may also be affected by the fact that New Brunswick, where most of our participants lived, had very low case counts during this time. Across the 2-week window of data collection, only 25 new cases were detected in the province (although all but one of these were related to a single outbreak in Campbellton). Despite the fact that most participants lived in the same province, restrictions were being relaxed across Canada, so we expected healthcare workers to have similar experiences during this time period.

**Figure 1.**
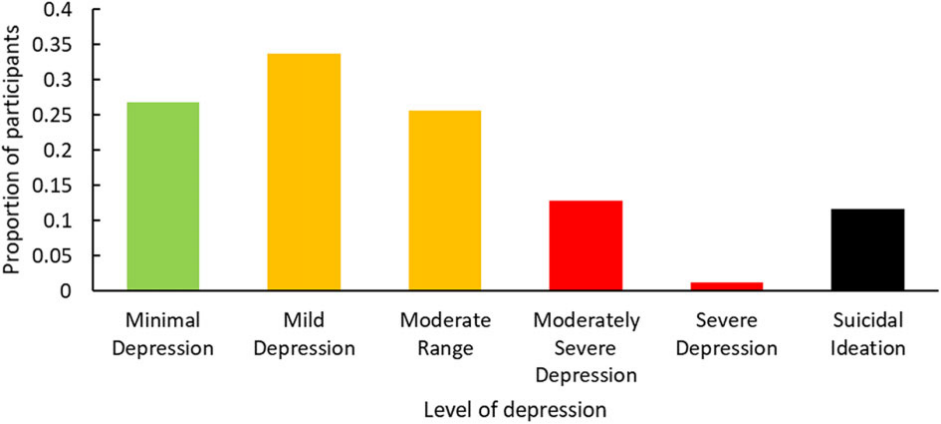
Number of participants with each level of depression based on the PHQ-9. The PHQ-9 is a self-administered test for initial assessment of depression and need for follow-up in patients. Although it is not a stand-alone diagnostic tool, it has a sensitivity of 88% and a specificity of 88% for major depression. Green bars indicate a low level of concern (PHQ-9 scores from 0-4). Yellow bars indicate recommendation of follow-up with mental health professional (PHQ-9 scores from 5-14). Red bars indicate the need for immediate treatment (PHQ-9 scores ≥15; see Kroenke et al.^32^). PHQ-9 indicates Patient Health Questionnaire-9.

The mean PHQ-9 score was 8.0 (SD = 5.3), corresponding to mild depression according to PHQ-9 guidelines.^32^ Although PHQ-9 scores cannot be used to make clinical diagnoses, guidelines also suggest that scores between 5 and 14 should be followed up with clinical interviews and individuals with scores greater than 14 should be immediately treated for depression. Figure 1 presents the proportion of participants who would be classified as having mild, moderate, and severe depression as well as those who reported suicidal ideation. Approximately 75% of participants had PHQ-9 scores greater than 5, suggesting the need for clinical follow-up. It is concerning that almost 50% of participants reported moderate to severe depression and approximately 10% reported suicidal ideation in the past week. These results highlight the need to assess the mental health of healthcare workers during this pandemic, even when case counts are low. The effects of psychological distress are cumulative; thus, in addition to increased infection control measures and communication, healthcare managers should support wellness in employees by providing resources to improve social support and individual resiliency^35^ and mental health support.^10^ Having chronic burnout can lead to clinical depression,^11^ and although our participants did not report significant burnout symptoms, it is possible that they did have symptoms during the earlier stages of the first COVID-19 wave. This causal link cannot be made based on current data, but it is possible that this contributed to the levels of depression observed.

These findings support previous research about psychological distress during health emergencies,^6–9^ which suggest that healthcare workers have mental health issues during acute pandemics that can have downstream effects related to employee satisfaction. Thus, in addition to the implementation of communication protocols, additional protective equipment and screening can alleviate some of the acute stressors present in a pandemic situation,^27,28^ supporting the importance of targeted mental health interventions to these employees.^14,15^

To better understand the connection between worker well-being and employer actions, Pearson correlations were conducted between PHQ-9 and GWB scores, number of measures being taken by employers, perceived satisfaction with employers’ actions, and perceived impacts of the pandemic on physical and mental health. Patient Health Questionnaire-9 scores were significantly correlated with self-assessment of negative physical health effects, *r* = .302, *P* = .005, and mental health effects, *r* = .528, *P* < .001. Interestingly, PHQ-9 scores were not significantly correlated with the number of safety measures being instituted by an employer.

General Well-Being Questionnaire burnout scores were negatively correlated with satisfaction with the safety measures put into place, *r* = −.273, *P* = .011, suggesting that greater satisfaction with measures is associated with less severe symptoms of burnout. General Well-Being Questionnaire stress scores were moderately correlated with satisfaction with implementation of safety measures, *r* = −.191, *P* = .079, although the association was not statistically significant. General Well-Being Questionnaire subscale scores were negatively correlated with satisfaction with employer communication (burnout: *r* = −.329, *P* = .002; stress: *r* = −.272, *P* = .011). Finally, both GWB scores were correlated with perception of effects of physical health (burnout: *r* = .430, *P* < .001; stress: *r* = .349, *P* = .001) and of mental health (burnout: *r* = .583, *P* < .001; stress: *r* = .483, *P* < .001). These findings highlight the need to treat the ongoing mental health issues amongst healthcare workers, to avoid the onset of burnout.^11,13^

### Limitations and future directions

The COVID-19 pandemic has caused increases in mental health concerns across society, and current results suggest that healthcare workers are no exception. Most of the respondents in this study had PHQ-9 scores indicating mild to moderate symptoms of depression that should be followed up with clinical assessment by a mental health professional. Future research should focus on assessing the specific chronic and acute stressors associated with mental health issues in healthcare workers in order to create interventions that are optimally implemented. We would suggest that future researchers could track the effectiveness of these interventions in preventing employee burnout and absenteeism.^7^ Work in this vein is already being conducted regionally,^13^ and it would be valuable to expand this to a national scale.

One of the main limitations of this study pertains to the participant sample—most participants came from the same province, which does limit the generalizability of results; however, we believe that the similar trend in active COVID-19 cases across Canada during the time the study was conducted does allow for some generalizability. Although future research could replicate this study with a larger, more national (or international) sample to confirm generalizability, we also wish to stress that the study was conducted in New Brunswick, a province in which COVID-19 case counts have been significantly below the national average throughout the pandemic, which one would expect to lead to a healthcare workforce with better mental health. Based on this expectation, the findings are even more shocking—if this level of mental health distress is present in healthcare workers in a relatively unaffected province, we should be very worried about what the situation may be in other provinces with higher caseload.

The current findings replicate research conducted during previous health emergencies, indicating that healthcare workers have mental health issues during times of acute pandemics, which can have knock-on effects on employee satisfaction.^6–9^ We are therefore adding to the argument presented in previous research that it is of utmost importance to provide targeted mental health interventions to these employees,^10,14,15^ which includes regular screening for symptoms of burnout and mental health decline, followed by clear and practical next steps, including paid time off, provision of mental healthcare, and peer support groups. Our findings also provide an impetus for action by health leaders and managers, from the higher administration of health authorities to individuals who work in wards in workplaces. Overall, our results indicate that employees can understand the policies implemented by their workplaces and appreciate pandemic-related employer communications. We encourage health leaders to communicate policy changes to employees and provide a clear rationale for changes. It is imperative that health leaders and managers implement ongoing screening and monitoring of employee mental health, particularly during times of acute stressors such as the current pandemic. Treatment of burnout is often more straightforward than the treatment of depression, and as such, the first goal for leaders should be to assess and treat burnout. This is also an effective use of resources as burnout is often a precursor to depression. An effective way to do this is to ensure workload is managed, even during times of acute stress, while also encouraging appropriate time off for employees. Combining such practices, along with opportunities for mental health education, allows the best possible mental health outcomes for employees.

## Conclusion

In a long-lasting pandemic, it is important to have interventions in place as early as possible and to keep them in place throughout the crisis. Our findings suggest that open and frequent communication with employees and the institution of appropriate protective measures can optimize the wellness of workers and prevent absenteeism during a healthcare crisis. Additionally, the provision of targeted mental health interventions may reduce the likelihood of burnout. These findings can be used to improve policies for the wellness of healthcare workers, both in Canada and throughout the world.
